# New Zealand cricket injury analysis based on 12 years of Accident Compensation Corporation data

**DOI:** 10.1136/bmjsem-2022-001340

**Published:** 2022-08-02

**Authors:** Sibi Walter, Doug King, Patria Hume

**Affiliations:** 1 Faculty of Health, University of Canterbury, Christchurch, New Zealand; 2 Traumatic Brain Injury Network (TBIN), Auckland University of Technology, Auckland, New Zealand; 3 Faculty of Health and Environmental Science, Sports Performance Research in New Zealand, Auckland University of Technology, Auckland, New Zealand

**Keywords:** cricket, epidemiology, female, injury, surveillance

## Abstract

**Objectives:**

To provide epidemiological data for cricket injuries in New Zealand.

**Methods:**

A retrospective analytical review using epidemiological cricket data obtained from the national Accident Compensation Corporation (ACC) for 2005–2016. Injury incidence was calculated per 1000 participants.

**Results:**

There were 86 562 injuries (77 212 males and 9350 females) during the 12 years with higher injury incidence for males (64.1) than females (36.1). While cricket-related injury claims increased by 42.6%, the injury incidence decreased from 59.0 in 2006 to 42.8 in 2016. The pooled injury rate per 1000 participants was highest for hand/fingers (9.2) and lumbar (8.1) body regions, and for contact (44.7) activities. Players aged 10–20 years were more likely to experience injury.

**Conclusions:**

Analysis of 12 years of ACC cricket-related injury claims showed only minimal reductions in injury incidence over the years. Therefore, cricket-related contact injuries to the hand/fingers and head need to be the focus of injury prevention programmes (eg, via promoting use of protective gear and correct technique), particularly in players aged 10–20 years.

**WHAT IS ALREADY KNOWN ON THIS TOPIC**

- Existing injury surveillance research in cricket has focused on elite cricketers.
- These studies have reported high prevalence of non-contact type injuries attributed to playing and training workload.

**WHAT THIS STUDY ADDS**

- The 12-year analysis of cricket injury data from our study revealed a high injury incidence of contact type injuries across all age groups and male and female participants.

**HOW THIS STUDY MIGHT AFFECT RESEARCH, PRACTICE OR POLICY**

- Cricket coaches at all levels of play need to emphasise use of protective gear and correct technique to reduce risk of contact type injuries.
- Injury prevention programmes focused on youth cricket players are needed.

## Introduction

Cricket is a popular summer sport traditionally played by Commonwealth nations.^1 2^ New Zealand (NZ)’s domestic outdoor cricket season runs from October to March. All age groups in the country play cricket, often formally starting in primary school, and progressing to recreational weekend cricket, with the pinnacle being elite first-class cricket. Globally, the introduction of the shorter franchise-based Twenty20 (T20) game format and internet streaming of cricket matches has coincided with increased spectator popularity and has likely contributed to larger participation numbers.^2^ In NZ, nationwide cricket participation numbers have increased by 75%, with 97 263 registered cricketers in the 2005–2006 season to 170 344 registered in the 2015–2016 season.^3^ With these sizeable public participation numbers, it is essential to monitor potential injury risks and ascertain if there is a widespread sports injury problem. The first stage in sports injury prevention is identifying the extent of the problem via quantification by conducting sports injury surveillance.^4 5^

Currently, there is no published nationwide study reporting injury incidence across all participation levels of cricket. In NZ, the last published cricket injury surveillance study was in 2008, and was focused solely on elite cricketers.^6^ Given the popularity of cricket in NZ, quantifying nationwide cricket injury incidence will help better understand cricket injury aetiology. Therefore, this study’s objective was to describe cricket-related injuries in the NZ population requiring medical treatment (as determined from the Accident Compensation Corporation (ACC) database) from 2005 to 2016.

## Methods

### Participants

NZ residents of all age groups who claimed medical treatment and rehabilitation costs from the ACC for a cricket-related injury from 2005 to 2016 were included in this study.

### Data collected

The NZ national cricket organisation does not capture nationwide cricket-related injury data across all levels of cricket, so data from the ACC database were obtained to describe the epidemiological extent of cricket-related injuries during the 12-year study period.

NZ’s ACC scheme provides registered medical practitioner information related to the patients’ injury diagnosis and medical care provided. Compensation covers costs towards medical treatment and rehabilitation.^7^ Details on the ACC injury reporting system and data are available in our series of papers.^8–13^

Acute personal injury claims,^14^ termed minor or moderate-to-serious claims (MSC) terms are defined under the Injury Prevention, Rehabilitation and Compensation Act, 2001 and identify ACC as responsible for providing costs of the injury claims lodged. For a claim to be classified as MSC, the injury typically requires assistance beyond medical treatment alone (ie, a combination of medical care, rehabilitation costs and income replacement for employment time lost because of the injury).^15^ This study included MSC claims from 1 January 2005 to 31 December 2016 that resulted in an injury from participation in cricket. No analyses were completed to identify multiple injuries per participant, so the number of participants is not provided.

### Injury definition

The injury definition for this study was ‘any injury (minor, moderate-to-serious and serious injury) that had been assessed and reported by a registered health practitioner as a result of sports participation’.^7^ To be included in the dataset for analysis, the ACC injury claim had to have been recorded as related to cricket.

### Pooled analysis

Injury incidence data were pooled^16 17^ to provide a more accurate injury incidence estimate.^18 19^ All data included in analysis needed to have similar definitions, have a comparable population and have adequacy and specificity of exposure data.^20^ This approach has been reported for rugby league injury epidemiological studies.^16 21^ An overall estimation of injuries was recorded by incorporating data provided by prior studies.^22^ Although there are limitations of a pooled analysis methodology,^18 23^ the strength is that it provides more accurate estimates of injury rates.^16 18^

### Statistics

ACC data were analysed by age group, injured body area (reclassified according to the Orchard sports injury and illness classification system), type and diagnosis. Injury causes were grouped into contact, non-contact and other mechanism of injury. Only new injury claims from 2005 to 2016 were considered; all previous injury claims data were removed from the data to calculate the injury incidence rate only for each calendar year’s original injury claims and pooled to provide an overall injury incidence rate.

All collected data entered into a Microsoft Excel spreadsheet were analysed with SPSS (released 2017, IBM SPSS Statistics for Windows, V.25.0, IBM, Armonk, New York, USA). Data were reported as means and 95% CIs,^24^ with an independent t-test used for comparisons. A one-sample χ^2^ test was used for comparison between reporting years for the number of claims. Injury incidence was calculated using participation data supplied by NZ cricket. Paired sampled t-tests were used to compare between male and female injury claims. Cohen’s d effect sizes were computed to complement interpretation of results, with effect sizes being interpreted as negligible/very small (d<0.20), small (d=0.20–0.49), medium (d=0.50–0.79) or large (d>0.80).^25^ Statistical significance was set at p<0.05.

### Patient and public involvement

Patients and/or the public were not involved in the design, or conduct, or reporting, or dissemination plans of this research.

## Results

There were 86 562 injuries (77 212 males and 9350 females) over the 12 years.

Results presented were analysed by sex-specific differences in injury incidence over the years, ages, injured body site and injury type.

### Years

There were more injury claims in 2016 for males (t_(17)_=−4.2; p=0.0006) and females (t_(17)_=−3.0; p=0.0073) compared with 2005 (table 1). Males recorded more claims than females (t_(11)_=27.5; p<0.0001) over the 12 years. There were meaningful differences in claim numbers over all years for males (t_(17)_=28.8; p<0.0001), females (t_(17)_=39.2; p<0.0001) and for all claims recorded (t_(17)_=29.9; p<0.0001). Although total claim numbers increased from 2005 to 2016 (5737 vs 8183; t_(17)_=−4.2; p=0.0005; d=0.31), the injury incidence decreased (59.0 (95% CI 57.5 to 60.5) vs 42.8 (95% CI 58.7 to 59.6) per 1000 participants).

**Table 1:** Injury claims by reporting years for total, mean claims per year with 95% CI, total registered participants and injury rate per 1000 participants by male, female and combined total for cricket in New Zealand from 2005 to 2016

Gender	Injury rate (95% CI)*	Total participants	Injuries per year Mean (95% CI)	Total injuries	Year
Males					
	2005	5044^acfghijklno^	280.2 (121.9 to 438.5)	N/A	–
	2006	5199^acfghijklmno^	288.8 (124.5 to 453.2)	85 215	59.2 (57.6 to 60.8)
	2007	6194^acdehlmno^	344.1 (148.9 to 539.3)	86 514	60.1 (58.5 to 61.8)
	2008	6343^acdehlmno^	352.4 (155.9 to 548.9)	88 517	70.0 (68.3 to 71.7)
	2009	6658^acdefgjno^	369.9 (165.8 to 574.0)	93 342	71.3 (69.6 to 73.1)
	2010	6484^acdejlmno^	360.2 (162.0 to 558.5)	95 603	67.8 (66.2 to 69.5)
	2011	5915^acdehiklmno^	328.6 (148.1 to 509.1)	93 743	63.1 (61.5 to 64.7)
	2012	6527^acdejlmno^	362.6 (161.4 to 563.8)	95 836	68.1 (66.5 to 69.8)
	2013	6901^acdefgijkn^	383.4 (176.0 to 590.8)	112 780	61.2 (59.8 to 62.7)
	2014	7033^acdefgijkn^	390.7 (180.0 to 601.4)	112 956	62.3 (60.8 to 63.7)
	2015	7619^acdefghijklm^	423.3 (198.2 to 648.4)	116 093	65.6 (64.2 to 67.1)
	2016	7295^acdefghijk^	405.3 (194.2 to 616.4)	125 316	58.2 (56.9 to 59.6)
	**Total†**	**77 212^ac^**	**6434.3 (5943.1 to 6925.6**)	**1 105 915**	**64.1 (63.6 to 64.6)**
Females					
	2005	693^abfghio^	38.5 (12.0 to 65.0)	N/A	–
	2006	651^abfghiklmno^	36.2 (13.4 to 59.0)	12 048	57.5 (53.4 to 62.0)
	2007	789^abde^	43.8 (17.5 to 70.2)	11 650	55.9 (51.7 to 60.3
	2008	774^abdeh^	43.0 (17.9 to 68.1)	11 831	66.7 (62.2 to 71.5)
	2009	846^abdegjk^	47.0 (22.5 to 71.5)	11 538	73.3 (68.5 to 78.4)
	2010	784^abde^	43.6 (18.3 to 68.8)	11 668	67.2 (62.6 to 72.1)
	2011	743^abho^	41.3 (16.9 to 65.6)	18 231	40.8 (37.9 to 43.8)
	2012	744^abeho^	41.3 (17.0 to 65.7)	15 993	46.5 (43.3 to 50.0)
	2013	782^abe^	43.4 (16.5 to 70.4)	25 964	30.1 (28.1 to 32.3)
	2014	781^abeo^	43.4 (16.4 to 70.4)	31 760	24.6 (22.9 to 26.4)
	2015	875^abe^	48.6 (19.0 to 78.2)	41 895	20.9 (19.5 to 22.3)
	2016	888^abdejkm^	49.3 (19.6 to 79.1)	45 028	19.7 (18.5 to 21.1)
	**Total†**	**9350^ab^**	**779.2 (735.5 to 822.8**)	**237 606**	**36.1 (35.3 to 36.9)**
Total					
	2005	5737^afghijklmno^	318.7 (135.1 to 502.3)	N/A	–
	2006	5850^afghijklmno^	325.0 (139.0 to 511.0)	97 263	59.0 (57.5 to 60.5)
	2007	6983^adehlmno^	387.9 (167.3 to 608.5)	97 913	59.7 (58.2 to 61.3)
	2008	7117^adehlmno^	395.4 (174.8 to 615.9)	100 348	69.6 (68.0 to 71.2)
	2009	7504^adefijno^	416.9 (189.1 to 644.7)	104 860	71.6 (70.0 to 73.2)
	2010	7268^adehjlmno^	403.8 (181.2 to 626.3)	107 271	67.8 (66.2 to 69.3)
	2011	6658^adehiklmno^	369.9 (166.9 to 572.8)	111 947	52.8 (51.5 to 54.2)
	2012	7271^adejlmno^	403.9 (180.2 to 627.7)	111 744	49.7 (48.6 to 59.8)
	2013	7683^adefgijkn^	426.8 (195.5 to 658.1)	138 744	49.7 (48.6 to 50.9)
	2014	7814^adefgijkno^	434.1 (199.6 to 668.7)	144 717	48.6 (47.5 to 49.7)
	2015	8494^adefghijklm^	471.9 (221.2 to 722.5)	157 988	48.2 (47.2 to 49.3)
	2016	8183^adefghijkm^	454.6 (216.4 to 692.8)	170 344	42.8 (41.9 to 43.8)
	**Total†**	**86 562**	**4809.0 (2174.2 to 7443.8)**	**1 343 521**	**59.1 (58.7 to 59.6)**

Significant difference (p<0.05) than (a)=over reporting years; (b)=male; (c)=female; (d)=2005; (e)=2006; (f)=2007; (g)=2008; (h)=2009; (i)=2010; (j)=2011; (k)=2012; (l)=2013; (m)=2014; (n)=2015; (o)=2016.

*Injury rate per 1000 registered participants.

†Pooled data.

### Age group

Males recorded more injuries across the study than females in all the age groups except the 80–84 (t_(11)_=−1.3; p=0.2118; d=0.53) and 85+ (t_(11)_=−1.3; p=0.2064; d=0.57) years age groups (table 2). Females recorded the most injuries in the 10–14 years age group (n=2168), whereas males recorded the most injuries in the 15–19 years age group (n=13 531).

**Table 2:** Injury claims for age groups for total, mean claims per year with 95% CI by male, female and combined total for cricket in New Zealand from 2005 to 2016

Age group	Male		Female		Total	
	Total injuries	Average injuries per year Mean (95% CI)	Total injuries	Average injuries per year Mean (95% CI)	Total injuries	Average injuries per year Mean (95% CI)
0–4	180^ac^	15.0 (11.1 to 18.9)	55^ab^	4.6 (3.4 to 5.8)	235^a^	19.6 (15.2 to 23.9)
5–9	2795^ac^	232.9 (212.3 to 253.5	542^ab^	45.2 (40.3 to 50.1)	3337^a^	278.1 (255.6 to 300.6)
10–14	12 941^ac^	1078.4 (1016.5 to 1140.3)	2168^ab^	180.7 (161.9 to 199.4)	15 109^a^	1259.1 (1182.4 to 1335.7)
15–19	13 531^ac^	1127.6 (1062.3 to 1192.9)	1830^ab^	152.5 (143.9 to 161.1)	15 361^a^	1280.1 (1213.7 to 1346.4)
20–24	10 282^ac^	856.8 (799.0 to 914.7)	1062^ab^	88.5 (83.9 to 93.1)	11 344^a^	945.3 (888.0 to 1002.7)
25–29	10 029^ac^	835.8 (759.1 to 912.4)	766^ab^	63.8 (55.4 to 72.3)	10 795^a^	899.6 (819.6 to 979.6)
30–34	7882^ac^	656.8 (582.3 to 731.4)	514^ab^	42.8 (38.1 to 47.5)	8396^a^	699.7 (623.5 to 775.9)
35–39	5964^ac^	497.0 (438.1 to 555.9)	589^ab^	49.1 (42.5 to 55.7)	6553^a^	546.1 (483.6 to 608.6)
40–44	5055^ac^	421.3 (370.1 to 472.4)	630^ab^	52.5 (46.0 to 59.0)	5685^a^	473.8 (418.6 to 528.9)
45–49	3825^ac^	318.8 (282.7 to 354.8)	479^ab^	39.9 (35.1 to 44.7)	4304^a^	358.7 (319.9 to 397.4)
50–54	2141^ac^	178.4 (157.6 to 199.2)	269^ab^	22.4 (18.5 to 26.4)	2410^a^	200.8 (177. to 224.4)
55–59	1201^ac^	100.1 (91.0 to 109.2)	141^ab^	11.8 (9.1 to 14.4)	1342^a^	111.8 (100.7 to 123.0)
60–64	715^ac^	59.6 (53.6 to 65.5)	117^ab^	9.8 (7.9 to 11.6)	832^a^	69.3 (62.4 to 76.3)
65–69	364^ac^	30.3 (24.9 to 35.8)	92^ab^	7.7 (5.8 to 9.5)	456^a^	38.0 (31.4 to 44.6)
70–74	181^ac^	15.1 (12.4 to 17.8)	49^ab^	4.1 (2.9 to 5.2)	230^a^	19.2 (15.5 to 22.8)
75–79	83^ac^	6.9 (4.1 to 9.7)	20^ab^	1.7 (0.7 to 2.6)	103^a^	8.6 (5.4 to 11.8)
80–84	28^a^	2.3 (1.4 to 3.3)	19^a^	1.6 (0.9 to 2.3)	47^a^	3.9 (2.8 to 5.0)
85+	15^a^	1.3 (0.5 to 2.0)	8^a^	0.7 (0.0 to 1.3)	23^a^	1.9 (1.0 to 2.9)

Significant difference (p<0.05) than (a)=over reporting years; (b)=male; (c)=female.

### Body site

Males recorded more injuries to the head/neck (Relative risk (RR) 1.23 (95% CI 1.17 to 1.29); p<0.0001; d=11.81), upper limb (RR 1.88 (95% CI 1.81 to 1.95); p<0.0001; d=8.73), lower limb (RR 1.73 (95% CI 1.67 to 1.79); p<0.0001; d=9.40) and chest/back/other (RR 2.36 (95% CI 2.23 to 2.49); p<0.0001; d=8.47) than females over the study (table 3). The upper limb sustained the most injuries (22.4 (95% CI 22.2 to 22.7) per 1000 participants) and the hand and fingers sustained the highest injury rate (9.2 (95% CI 9.0 to 9.3) per 1000 participants) for injury site.

**Table 3:** Injury claims by and type for total, mean claims per year with 95% CI and pooled injury rate per 1000 participants by male, female and combined total for cricket in New Zealand from 2005 to 2016

	Male			Female			Total		
	Total no	Average claims per year Mean (95% CI)	Injury rate (95% CI)*	Total no	Average claims per year Mean (95% CI)	Injury rate (95% CI)*	Total no	Average claims per year Mean (95% CI)	Injury rate (95% CI)*
**Injury site**									
**Head/Neck**	**10 267^acefg^**	**855.6 (802.7 to 908.4)**	**9.3 (9.1 to 9.5)**	**1793^abefg^**	**149.4 (139.8 to 159.0)**	**7.5 (7.2 to 7.9)**	**12 060^aefg^**	**1005.0 (945.7 to 1064.3)**	**9.0 (8.8 to 9.1)**
Head	8192	682.7 (633.8 to 731.5)	7.4 (7.2 to 7.6)	1433	119.4 (111.4 to 127.4)	6.0 (5.7 to 6.4)	9625	802.1 (748.0 to 856.2)	7.2 (7.0 to 7.3)
Neck	2075	172.9 (152.7 to 193.1)	1.9 (1.8 to 2.0)	360	30.0 (24.6 to 35.4)	1.5 (1.4 to 1.7)	2435	202.9 (178.6 to 227.2)	1.8 (1.7 to 1.9)
**Upper limb**	**27 053^acdfg^**	**2254.4 (2049.6 to 2459.2)**	**24.5 (24.2 to 24.8)**	**3087^abdg^**	**257.3 (240.7 to 273.8)**	**13.0 (12.5 to 13.5**)	**30 140^adfg^**	**2511.7 (2292.9 to 2730.5)**	**22.4 (22.2 to 22.7)**
Shoulder	8636	719.7 (633.6 to 805.7)	7.8 (7.6 to 8.0)	821	68.4 (60.0 to 76.8)	3.5 (3.2 to 3.7)	9457	788.1 (695.4 to 880.8)	7.0 (6.9 to 7.2)
Upper and lower limb	2153	179.4 (159.1 to 199.7)	1.9 (1.9 to 2.0)	304	25.3 (21.2 to 29.5)	1.3 (1.1 to 1.4)	2457	204.8 (181.9 to 227.6)	1.8 (1.8 to 1.9)
Elbow	1085	90.4 (82.6 to 98.2)	1.0 (0.9 to 1.0)	141	11.8 (10.1 to 13.4)	0.6 (0.5 to 0.7)	1226	102.2 (93.7 to 110.7)	0.9 (0.9 to 1.0)
Wrist	4085	340.4 (314.8 to 366.1)	3.7 (3.6 to 3.8)	603	50.3 (44.7 to 55.8)	2.5 (2.3 to 2.7)	4688	390.7 (363.8 to 417.5)	3.5 (3.4 to 3.6)
Hand and fingers	11 094	924.5 (844.4 to 1004.6)	10.0 (9.8 to 10.2)	1218	101.5 (94.5 to 108.5)	5.1 (4.85 to 5.4)	12 312	1026.0 (943.2 to 1108.8)	9.2 (9.0 to 9.3)
**Lower limb**	**25 159^acdeg^**	**2096.6 (1922.1 to 2271.1)**	**22.7 (22.5 to 23.0)**	**3126^abdg^**	**260.5 (242.2 to 278.8)**	**13.2 (12.7 to 13.6)**	**28 285^adeg^**	**2357.1 (2167.5 to 2546.7)**	**21.1 (20.8 to 21.3)**
Hip, thigh	5656	471.3 (428.9 to 513.7)	5.1 (5.0 to 5.2)	504	42.0 (36.6 to 47.4)	2.1 (1.9 to 2.3)	6160	513.3 (467.1 to 559.5)	4.6 (4.5 to 4.7)
Knee	8272	689.3 (631.5 to 747.2)	7.5 (7.3 to 7.6)	998	83.2 (78.1 to 88.2)	4.2 (3.9 to 4.5)	9270	772.5 (712.5 to 832.5)	6.9 (6.8 to 7.0)
Lower leg	2822	235.2 (209.6 to 260.7)	2.6 (2.5 to 2.6)	384	32.0 (27.5 to 36.5)	1.6 (1.5 to 1.8)	3206	267.2 (239.6 to 294.7)	2.4 (2.3 to 2.5)
Ankle	5405	450.4 (412.3 to 488.5)	4.9 (4.8 to 5.0)	832	69.3 (63.1 to 75.6)	3.5 (3.3 to 3.7)	6237	519.8 (476.7 to 562.8)	4.6 (4.5 to 4.8)
Foot	3004	250.3 (231.0 to 269.7)	2.7 (2.6 to 2.8)	408	34.0 (30.4 to 37.6)	1.7 (1.6 to 1.9)	3412	284.3 (262.6 to 306.1)	2.5 (2.5 to 2.6)
**Chest/Back/Other**	**14 733^acdef^**	**1227.8 (1109.7 to 1345.8)**	**13.3 (13.1 to 13.5)**	**1344^abdef^**	**112.0 (103.1 to 120.9)**	**5.7 (5.4 to 6.0)**	**16 077^adef^**	**1339.8 (1217.2 to 1462.3)**	**12.0 (11.8 to 12.2)**
Chest	1830	152.5 (143.3 to 161.7)	1.7 (1.6 to 1.7)	158	13.2 (11.7 to 14.6)	0.7 (0.6 to 0.8)	1988	165.7 (155.9 to 175.5)	1.5 (1.4 to 1.5)
Abdomen	767	63.9 (55.1 to 72.7)	0.7 (0.6 to 0.7)	62	5.2 (3.7 to 6.6)	0.3 (0.2 to 0.3)	829	69.1 (59.8 to 78.3)	0.6 (0.6 to 0.7)
Lumbar	10 022	835.2 (724.4 to 946.0)	9.1 (8.9 to 9.2)	865	72.1 (64.3 to 79.8)	3.6 (3.4 to 3.9)	10 887	907.3 (791.4 to 1023.1)	8.1 (8.0 to 8.3)
Unknown	2114	176.2 (161.5 to 190.8)	1.9 (1.8 to 2.0)	259	21.6 (16.9 to 26.3)	1.1 (1.0 to 1.2)	2373	197.8 (180.1 to 215.4)	1.8 (1.7 to 1.8)
**Injury type**									
Soft tissue	59 746^ac^	3734.1 (2149.3 to 5319.0)	54.0 (53.6 to 54.5)	7174^ab^	448.4 (285.4 to 611.3)	30.2 (29.5 to 30.9)	66,920^a^	4182.5 (2445.0 to 5920.0)	49.8 (49.4 to 50.2)
Fracture/Dislocation	8250^c^	515.6 (28.4 to 1059.6)	7.5 (7.3 to 7.6)	794^ab^	49.6 (5.5 to 93.8)	3.3 (3.1 to 3.6)	9044	565.3 (22.4 to 1152.9)	6.7 (6.6 to 6.9)
Laceration/Puncture	5599^ac^	349.9 (62.0 to 637.8)	5.1 (4.9 to 5.2)	603^ab^	37.7 (6.0 to 69.4)	2.5 (2.3 to 2.7)	6,202^a^	387.6 (68.3 to 707.0)	4.6 (4.5 to 4.7)
Dental injury	2347	146.7 (66.0 to 459.3)	2.1 (2.0 to 2.2)	481	30.1 (4.0 to 94.1)	2.0 (1.9 to 2.2)	2828	176.8 (20.0 to 553.5)	2.1 (2.0 to 2.2)
Other	1714	107.1 (89.3 to 303.6)	1.5 (1.5 to 1.6)	214	13.4 (9.5 to 36.3)	0.9 (0.8 to 1.0)	1928	120.5 (98.7 to 339.7)	1.4 (1.4 to 1.5)
Concussion	424	26.5 (30.0 to 83.0)	0.4 (0.3 to 0.4)	75	4.7 (5.3 to 14.7)	0.3 (0.3 to 0.4)	499	31.2 (5.3 to 97.7)	0.4 (0.3 to 0.4)
Gradual inflammation	132	8.3 (1.4 to 17.9)	0.1 (0.1 to 0.1)	9	0.6 (0.0 to 1.1)	0.0 (0.0 to 0.1)	141	8.8 (1.3 to 18.9)	0.1 (0.1 to 0.1)
**Injury cause**									
Contact	53 283^acij^	3330.2 (1850.1 to 4810.2)	48.2 (47.8 to 48.6)	6807^abij^	425.4 (221.4 to 629.5)	28.6 (28.0 to 29.3)	60 090^aij^	3755.6 (2082.8 to 5428.5)	44.7 (44.4 to 45.1)
Non-contact	23 536^achj^	1,.471.0 (515.8 to 2426.2)	21.3 (21.0 to 21.6)	2468^abhj^	154.3 (69.7 to 238.8)	10.4 (10. to 10.8)	26 004^ahj^	1625.3 (587.4 to 2663.1)	19.4 (19.1 to 19.6)
Other	393^achi^	24.6 (8.0 to 41.1)	0.4 (0.3 to 0.4)	75^abhi^	4.7 (0.2 to 9.2)	0.3 (0.3 to 0.4)	468^ahi^	29.3 (8.4 to 50.1)	0.3 (0.3 to 0.4)

Significant difference (p<0.05) than (a)=reporting years (b)=male; (c)=female; (d)=head/neck; (e)=upper limb; (f)=lower limb; (g)=chest/back/other; (h)=contact; (i)=non-contact; (j)=other.

*Pooled data.

†Injury rate per 1000 registered participants.

### Injury type

There were notable differences in the number of soft tissue injuries recorded over the study for males (3734.1 (95% CI 2149.3 to 5319.0); t_(15)_=5.0; p=0.0002), females (448.4 (95% CI 285.4 to 611.3); t_(11)_=5.9; p<0.0001) and total (4182.5 (2445.0 to 5920.0); t_(11)_=5.1; p=0.0001) injury claims recorded.

### Cause of injury

There were more injuries recorded due to contact than non-contact for males (RR 2.26 (95% CI 2.23 to 2.30); p=0.0344; d=0.80), females (RR 2.76 (95% CI 2.63 to 2.89); p=0.0215; d=0.92) and total (RR 2.31 (95% CI 2.28 to 2.34); p=0.0319; d=0.82) injury claims.

## Discussion

This study provided an epidemiological description of cricket-related injuries among the NZ resident population from the years 2005 to 2016. The key findings of this study were: (1) although injury claims increased, the incidence of injuries decreased; (2) males recorded more injuries than females in most age groups; (3) the upper limb sustained the most injuries and the hand and fingers sustained the highest injury rate and (4) there were more injuries due to contact than non-contact actions.

### Years

The number of cricket-related injury claims increased by 42.6% but the injury incidence decreased from 59% to 42.8% over the 12 years. The introduction of the T20 cricket format along with the availability of streaming international cricket matches may have caused a surge in the popularity of the sport and might have led to larger participation numbers. The introduction of shorter cricket competitions across different cricket levels such as club cricket, school cricket, regional cricket and metro cricket might have caused people to play more cricket. While it is hard to discern the cause of the 42.8% increase in injury claims, there was a related increase in cricket participation across NZ with a 47% increase with 85 215 people registered to play in 2005 and 125 316 registered to play in 2016.

### Age group

Adolescent athletes can encounter more epiphysial and stress-related injuries due to the undergoing hormonal changes which might then predispose them to acute injuries.^26^ The opportunity to play cricket at various levels such as school cricket, club cricket, recreational weekend cricket and regional age-group cricket may contribute to an increased volume of cricket participation in this age group. More frequent participation and training can improve skill development and provide opportunities for some to compete in representative teams at a regional or national level. It should be noted that injury onset at a younger age will affect performance and may limit further participation in the sport.^5^ Hence, monitoring this injury-prone age group of 10–19 years and implementing early preventative strategies is strongly recommended.

### Body site

Reviewing the injuries by body areas across the 12 years has highlighted that the injury prone areas differ between male and female participants. A higher incidence of hand injuries has also been reported among elite cricketers.^6 27 28^ It may be that contact and impact with the ball or falling with an outstretched arm while fielding all contribute to impact-related injuries at all levels of cricket. Injury incidence specific to body areas between elite cricketers and recreational cricketers may therefore differ.

### Injury type

The prevalence of impact-contact injuries to the hand and wrist area (eg, fractures and dislocations) are likely due to an improper technique employed while catching the ball, or potentially due to the absence of effective protective equipment when fielding. This may be due to the requirement that the only hand protection equipment allowed in cricket are wicket-keeping and batting gloves.

Contact injuries in cricket could be sustained due to impacts with balls, objects marking the boundaries or other equipment related to the game.^29^ The injuries recorded in the study were classified as either of contact or non-contact nature. In the current study, contact injuries were mostly due to impact with an object, the ground or a person and non-contact injuries are mainly due to repetitive strenuous movement.

In the upper limb, the hand and fingers may experience more contact type injuries, whereas the shoulder may sustain more non-contact type injuries. As non-contact injury type is mainly due to repetitive strenuous movement, the shoulder was most likely to have been injured due to repetitive movement in cricket.^30^ The repetitive strenuous shoulder movement is likely to have been due to a combination of throwing and bowling.

Unlike hand injuries, two-thirds of all lumbar injuries were of a non-contact nature. A commonly cited reason for elite cricketers’ lumbar injuries has been increased playing/training workload.^31^ Non-contact lower back soft tissue injuries occur mostly in the lumbar muscles and intervertebral discs, ‘as’ injury surveillance among South African provincial age-group cricketers revealed lower back muscle strains (78 injuries) were higher than stress fractures (33 injuries).^32^

Participants in this study included a nationwide population with data including primary school level cricketers to elite first-class cricketers. Therefore, an acute lumbar injury could have occurred to a recreational cricketer due to improper warmup or to an elite first-class cricketer due to training/bowling workload or vice versa.

While conducting injury surveillance, it is essential to record the level of the player given that elite players are likely to have access to a lot of injury prevention support, whereas recreational cricket players may not have access to or use protective equipment frequently.

Elite cricketers undergo intense workload, playing frequent matches and training several times per week, increasing their predisposition to overuse injuries.^33^ By contrast, recreational cricketers may not partake in regular warmups, may forego conditioning sessions and may just play cricket during the weekends, exposing them to sudden loading and greater risks of acute injuries. Therefore, elite cricketers may not experience impact-related face or finger injuries^34^ as often as recreational cricketers. However, elite cricketers may encounter greater overuse-related injuries.^35 36^ Therefore, elite cricketers’ injury surveillance may reveal a higher incidence of overuse injuries, whereas impact injuries may have a higher incidence among a nationwide cricketing population. Hence, while conducting cricket injury surveillance, it is critical to question the nature of the reported injury. Generalising injury aetiology of elite cricketers to a nationwide cricketing population may not always be applicable.

Data obtained from ACC did not reveal the injury-specific medical diagnosis; if such information was available, the nature of injuries could be classified as either acute or overuse. If injury onset data were available, it would provide insight into whether bowling, batting or fielding was a predominant factor for injury, and it would highlight the most injury-prone playing positions. Some cricket injury data may not have been recorded if the injury was not severe enough to qualify for an injury entitlement claim. Some individuals might not have sought medical care, which is a limitation to the current study.

## Conclusions

This study provided evidence that as cricket participation numbers increased there was a substantial increase in cricket-related injury claims. Analysis of 12 years of ACC cricket-related injury claims showed only minimal reductions in injury incidence over the years. Injury prevention programmes for cricket need to target the prevention of hand and finger, and head injuries particularly in players aged 10–20 years.

## Data Availability

The data that support the findings of this study are available on request.
